# *Odoribacter splanchnicus* bacteremia secondary to acute appendicitis: a case report with review of literature

**DOI:** 10.1093/jscr/rjae328

**Published:** 2024-05-24

**Authors:** Sreethish Sasi, Arun Prabhakaran Nair, Sanjay Doiphode, Tejeswi Shashidhar Gutti, Jouhar Kolleri, Muna Al-Maslamani

**Affiliations:** Infectious Diseases Division, Department of Internal Medicine, Hamad Medical Corporation, Doha 3050, Qatar; Infectious Diseases Division, Department of Internal Medicine, Communicable Diseases Center, Hamad Medical Corporation, Doha 3050, Qatar; Infectious Diseases Division, Department of Internal Medicine, Hamad Medical Corporation, Doha 3050, Qatar; Infectious Diseases Division, Department of Internal Medicine, Communicable Diseases Center, Hamad Medical Corporation, Doha 3050, Qatar; Microbiology Division, Department of Laboratory Medicine and Pathology, Hamad Medical Corporation, Doha 3050, Qatar; Department of Surgery, Hazem Mubaireek General Hospital, Hamad Medical Corporation, Doha 3050, Qatar; Department of Clinical Imaging, Hamad Medical Corporation, Doha 3050, Qatar; Infectious Diseases Division, Department of Internal Medicine, Hamad Medical Corporation, Doha 3050, Qatar; Infectious Diseases Division, Department of Internal Medicine, Communicable Diseases Center, Hamad Medical Corporation, Doha 3050, Qatar

**Keywords:** Odoribacter splanchnicus, Bacteroides, bacteremia, appendicitis

## Abstract

This report describes a rare instance of *Odoribacter splanchnicus* bacteremia secondary to acute appendicitis in a young man. Initially presenting with symptoms typical of appendicitis, he was diagnosed through clinical examination, laboratory tests, and computed tomography imaging, which confirmed an inflamed appendix with sealed perforation and abscess. *O. splanchnicus*, a Gram-negative anaerobe commonly found in the human gut, was identified as the causative agent through blood culture. The patient underwent successful laparoscopic appendectomy and was treated with intravenous amoxicillin–clavulanate, leading to a full recovery. This case highlights the potential of *O. splanchnicus* to act as an opportunistic pathogen in the context of intra-abdominal inflammation. It underscores the diagnostic challenges posed by *O. splanchnicus*, and the efficacy of advanced diagnostic tools like matrix-assisted laser desorption/ionization-time of flight mass spectrometry in identifying such rare infections.

## Introduction

*Odoribacter splanchnicus*, previously classified as *Bacteroides splanchnicus*, is a Gam-negative, strictly anaerobic bacterium commonly found in the human intestinal microbiota [1]. It belongs to the family *Odoribacteraceae* within the order *Bacteroidales* [2]. Known for its capacity to produce short-chain fatty acids (SCFAs), particularly butyrate, *O. splanchnicus* plays a vital role in maintaining gut homeostasis and has been associated with various health benefits [3]. However, alterations in its abundance have been linked to several disorders, including inflammatory bowel disease, non-alcoholic fatty liver disease, and cystic fibrosis. Additionally, recent studies suggest a potential role of *O. splanchnicus* in metabolic health, with associations observed between its abundance and parameters such as systolic blood pressure and lipid metabolism [1]. Despite its potential health-promoting effects, the specific interactions between *O. splanchnicus* and the host remain largely unexplored.

## Case presentation

**Figure 1:**
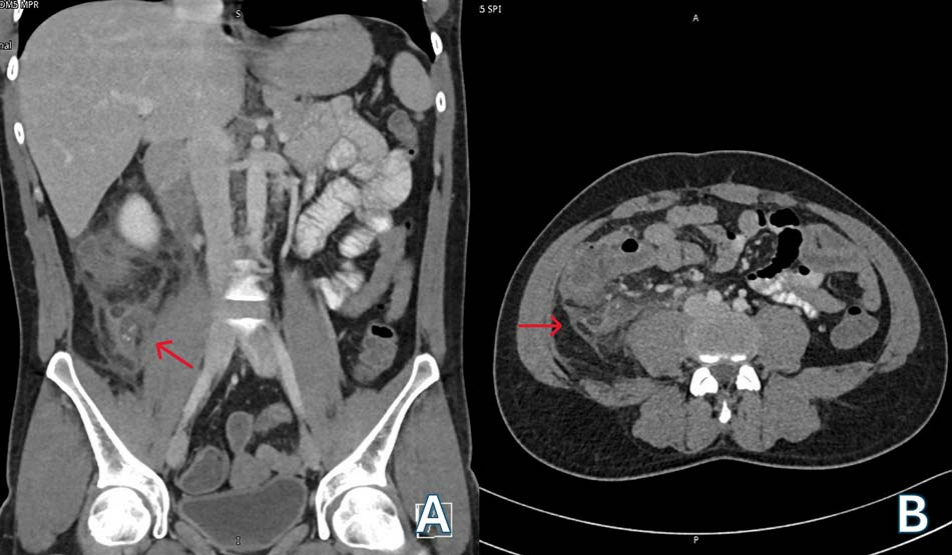
(A) Venous phase computed tomography (CT) of abdomen showing dilated appendix with peri appendicular fat stranding and inflammation (red arrow); (B) Axial computed tomography (CT) of abdomen showing retrocecal appendix with severe inflammation involving up to the retroperitoneal musculature (red arrow).

**Figure 2:**
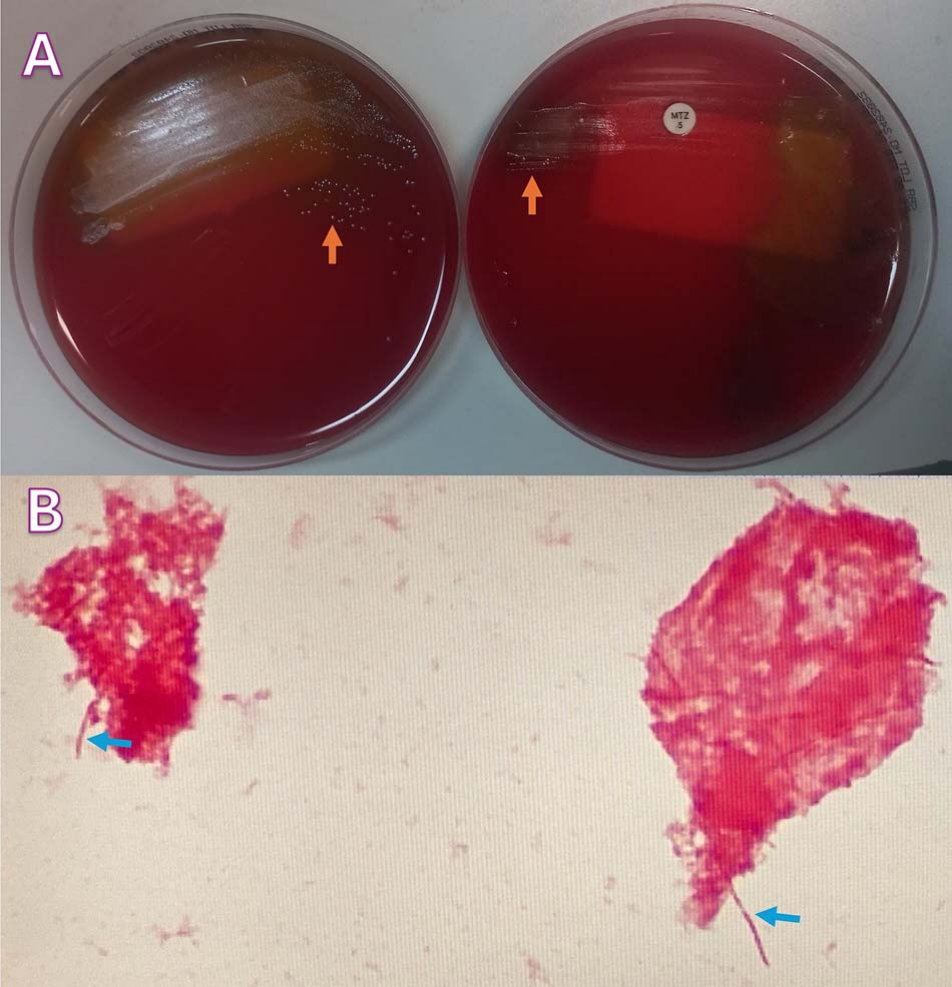
(A) Blood agar plates under anaerobic conditions growing moist and gray-white colonies (orange arrows); (B) Gram-stain analysis showing Gram-negative rods (blue arrows).

**Figure 3:**
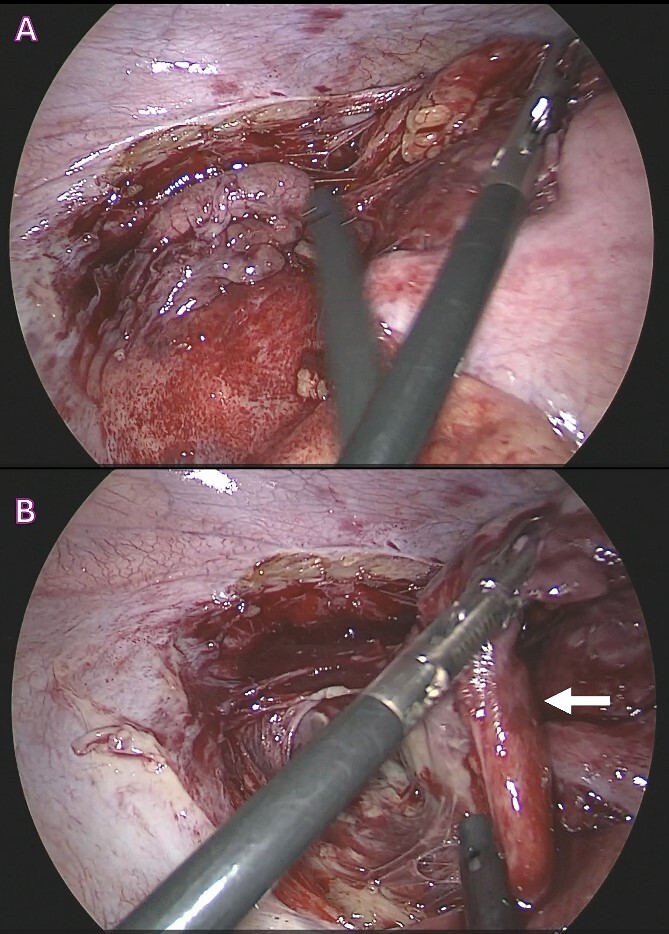
Surgical images during laparoscopic appendectomy showing; (A) acutely inflamed retrocecal appendix buried retroperitoneally; (B) retroperitoneal large abscess pocket with ruptured appendix with thick pus and inflammatory exudates (white arrow).

A 24-year-old Bangladeshi male presented to our emergency department with a 4-day history of right iliac fossa pain and fever. The patient denied experiencing diarrhea, vomiting, upper respiratory tract infection symptoms, radiation of pain, anorexia, testicular pain, or constipation. On examination, his temperature was 38.7°C, heart rate 108 bpm, blood pressure 116/66 mmHg, respiratory rate 18 breaths per minute, and oxygen saturation 100% in room air. Abdominal examination revealed localized tenderness and guarding in the right iliac fossa, along with positive rebound tenderness and Rovsing’s sign. Psoas sign and obturator sign were also elicited. Differential diagnoses included acute appendicitis, among other causes of abdominal pain, such as gastroenteritis, mesenteric lymphadenitis, incarcerated inguinal hernia, acute diverticulitis (less common in younger individuals), and pelvic inflammatory disease (less common in males). Investigations comprised laboratory tests, revealing elevated white blood cell count (14.1 × 10^3^/μl) and C-reactive protein (CRP) levels (228.2 mg/L), and imaging via Computed Tomography (CT) abdomen and pelvis, which confirmed an acutely inflamed retrocecal appendix with a suspicious sealed perforation and an abscess cavity behind the cecum (Fig. 1). Blood cultures were collected in two anaerobic and two aerobic bottles at the time of admission. These bottles were placed in a BacT/Alert system (Becton Dickinson, NJ) for incubation. On Day 3, tests from both anaerobic blood culture bottles came back positive, at 52 and 59.5 hours, respectively, and gram stain analysis revealed the presence of Gram-negative bacteria (Fig. 2B) when observed under a microscope. Specimens were then grown on common blood agar plates under anaerobic conditions at 35°C. On Day 5 after presentation, moist and gray-white colonies were found (Fig. 2A). These were rapidly identified as *O. splanchnicus* using matrix-assisted laser desorption/ionization-time of flight mass spectrometry (MALDI-TOF MS) Biotyper (Library BDAL-11897-4274 Version 12, Bruker Daltonics, Bremen, Germany) on the same day. Treatment involved laparoscopic appendectomy (Fig. 3) and subsequent administration of intravenous amoxicillin–clavulanate 1.2 g three times a day for 10 days. The patient responded well to treatment, with normalization of laboratory values (discharge CRP: 67.7mg/L) and resolution of fever. Blood culture repeated after 72 h of intravenous antibiotics was negative.

## Discussion

*O. splanchnicus* bacteremia secondary to appendicitis presents a unique clinical scenario, highlighting the potential for this commensal bacterium to become an opportunistic pathogen in the setting of intra-abdominal inflammation [4, 5]. Our case adds to the limited literature on such occurrences, underscoring the importance of considering unusual pathogens in the context of infectious complications following surgical procedures. This is only the third case report on this organism in literature. A comparative analysis of the three reports is provided in Table 1. Although *O. splanchnicus* is commonly found in the human gut microbiome, its role as a pathogen in appendicitis-associated bacteremia is rare and warrants further investigation. The association between *O. splanchnicus* bacteremia and appendicitis raises questions about the mechanisms by which this bacterium gains access to the bloodstream. It is plausible that disruption of the intestinal barrier integrity due to appendiceal inflammation allows translocation of *O. splanchnicus* from the gut lumen into the bloodstream [2, 4–6]. Additionally, factors such as impaired immune response and bacterial virulence properties may contribute to the development of bacteremia in the context of appendicitis [4]. Further studies elucidating the pathogenic mechanisms of *O. splanchnicus* in appendicitis-associated bacteremia are necessary to better understand its clinical significance and guide therapeutic interventions.

**Table 1:** Table comparing our cases with two previously reported cases in literature.

**Aspect**	**Case 1**	**Case 2**	**Case 3**
Author	Our case	Aoi Kanematsu *et al.* (Anaerobe, 2022)^4^	Hualiang Xiao *et al.* (Heliyon, 2024)^7^
Patient demographics	24-year-old Bangladeshi male	49-year-old Japanese male	29-year-old Chinese male
Initial presentation	Right iliac fossa pain, fever	Periumbilical colic pain, nausea	Lower abdominal pain, fever
Vital signs	Fever (38.7°C), tachycardia	Fever (37.2 °C), hypertension	Fever (36.8 °C), normal blood pressure
Laboratory findings	Elevated WBC (14.1 × 10^3^/μl), elevated CRP	Elevated WBC (13.0 × 10^3^/μl), elevated CRP	Elevated WBC (16.62 × 10^3^/μl), elevated neutrophils and CRP
Imaging	CT abdomen and pelvis with intravenous contrast	CT abdomen with intravenous contrast	CT abdomen and pelvis with intravenous contrast
Diagnosis	Acute gangrenous appendicitis	Suppurative appendicitis	Intestinal perforation
Blood culture	*O. splanchnicus* bacteremia	*O. splanchnicus* bacteremia	*O. splanchnicus* bacteremia
Antibiotic therapy	Amoxicillin–clavulanate	Cefmetazole switched to ampicillin–sulbactam	Cefoperazone–sulbactam, then imipenem–cilastatin
Surgical intervention	Laparoscopic appendectomy	Laparoscopic appendectomy	Emergency surgery for intestinal perforation
Outcome	Normalization of lab values, resolution of fever	Discharged on oral amoxicillin–clavulanate	Discharged after recovery from surgery

Diagnostic challenges posed by *O. splanchnicus* infections are evident, as traditional culture-based methods may lead to misidentification or underreporting of this pathogen. Advanced techniques such as MALDI-TOF MS [7] and 16S rRNA [5] sequencing are crucial for accurate identification, highlighting the importance of incorporating these methodologies in clinical practice. Our case highlights the utility of MALDI-TOF MS in rapidly confirming the identity of *O. splanchnicus*, thereby facilitating appropriate management strategies. Moving forward, heightened clinical awareness, coupled with advanced diagnostic methodologies, will enhance our ability to recognize and manage *O. splanchnicus* bacteremia secondary to appendicitis, ultimately improving patient outcomes [2, 5, 7].

Furthermore, our case underscores the importance of tailored antibiotic therapy based on susceptibility testing, as different cases may require varying treatment approaches. Continued research efforts and improved diagnostic techniques will enhance our understanding of *O. splanchnicus* pathogenesis and clinical significance, ultimately guiding more effective management strategies for patients with *O. splanchnicus*-associated infections.

Our case of *O. splanchnicus* bacteremia underscores the rarity and potential pathogenicity of this bacterium in human infections. Similar to previous reports, our patient’s presentation highlights the diverse clinical manifestations associated with *O. splanchnicus*, ranging from acute appendicitis to bloodstream infections. Despite its prevalence as a commensal in the human gut microbiome, *O. splanchnicus* has been sporadically implicated in various disease states, including appendicitis, peritonitis, and bacteremia, emphasizing the need for heightened clinical suspicion and accurate diagnosis.
