# Correction to: *Odoribacter splanchnicus* bacteremia secondary to acute appendicitis: a case report with review of literature

**DOI:** 10.1093/jscr/rjae416

**Published:** 2024-06-11

This is a correction to: Sreethish Sasi, Arun Prabhakaran Nair, Sanjay Doiphode, Tejeswi Shashidhar Gutti, Jouhar Kolleri, Muna Al-Maslamani, *Odoribacter splanchnicus* bacteremia secondary to acute appendicitis: a case report with review of literature, *Journal of Surgical Case Reports*, Volume 2024, Issue 5, May 2024, rjae328, https://doi.org/10.1093/jscr/rjae328

In the originally published version of the manuscript there were two errors in the legend to Figure 1. This should read: “(A) Venous phase computed tomography (CT) […]” and “(B) Axial computed tomography (CT) […]” instead of: “(A) Triphasic computed tomography (CT) […]” and “(B) sagittal computed tomography (CT) […]”.

The emendations have been made to the article.

